# Cross-sectional study on risk factors for Porcine Reproductive and Respiratory Syndrome virus sow herd instability in German breeding herds

**DOI:** 10.1186/s13028-018-0411-7

**Published:** 2018-09-19

**Authors:** Christina Nathues, Eltje Janssen, Andrea Duengelhoef, Heiko Nathues, Elisabeth grosse Beilage

**Affiliations:** 1 Veterinary Public Health Institute, Department of Clinical Research & Veterinary Public Health, Vetsuisse Faculty, University of Bern, Schwarzenburgstrasse 155, 3097 Liebefeld, Switzerland; 2 Federal Food Safety and Veterinary Office, Schwarzenburgstrasse 155, 3003 Bern, Switzerland; 3 Field Station for Epidemiology, University of Veterinary Medicine Hannover, Buescheler Strasse 9, 49456 Bakum, Germany; 4 Clinic for Swine, Department of Clinical Veterinary Medicine, Vetsuisse Faculty, University of Bern, Bremgartenstrasse 109a, 3012 Bern, Switzerland

**Keywords:** Herd management, Herd prevalence, PRRS control, Risk factors

## Abstract

**Background:**

Porcine Reproductive and Respiratory Syndrome virus (PRRSV) stability, besides meeting production targets, is also a requirement for several control options of PRRS in pig breeding farms. This study aimed to investigate the frequency of, and risk factors associated with, PRRSV instability in pig breeding farms in northern Germany. In 120 sow herds, a questionnaire on production and management parameters was filled, and blood samples from 30 suckling pigs from 10 different litters were taken and examined using real-time polymerase chain reaction (PCR).

**Results:**

PRRSV was detected in 32 herds (27%), thus classified as PRRSV-unstable. According to multivariable logistic regression analysis, a suckling period ≤ 21 days, a low distance between the cadaver collection site and the actual sow barn, ≥ 2 pig herds in a 1000 m radius, presence of external employees, a time interval between purchase of gilts of ≤ 9 weeks and a 1- or 2-weekly farrowing rhythm were associated with a higher risk of PRRSV instability.

**Conclusions:**

External and internal biosecurity as well as management factors were associated with PRRSV instability, which could be targeted by farmers and veterinarians to help them to achieve PRRSV PCR-negative status and in the control of PRRS.

**Electronic supplementary material:**

The online version of this article (10.1186/s13028-018-0411-7) contains supplementary material, which is available to authorized users.

## Background

Pig herds infected with Porcine Reproductive and Respiratory Syndrome virus (PRRSV) often develop corresponding clinical diseases in sows, boars and/or their offspring. It has been shown that the frequency of disease outbreaks and the severity of clinical symptoms in a herd are driven by virulence of the particular PRRSV isolate and risk factors, such as co-infections or lack of gilt acclimatisation [1, 2]. Vaccination against the Porcine Reproductive and Respiratory Syndrome (PRRS) is possible and commonly applied [3], but does not always sufficiently prevent clinical diseases [4], neither does it offer the possibility of elimination of the virus at farm level [5].

Taking these limitations of PRRS control by vaccination into account, additional measures are necessary. In order to control PRRS in such herds, different options are available at farm level, including close and rollover and test and removal [6–9]. However, these two options require herd stability in terms of (non-existent) PRRSV transmission from dams to their offspring. They usually work sufficiently only if no further vertical transmission of PRRSV from sows to suckling pigs occurs and only PRRSV negative piglets are weaned. Therefore, appropriate evaluation of the breeding herd PRRSV status and assessment of potential risk factors influencing it are important. According to Holtkamp’s definition, PRRSV stability [10] is the absence of virus circulation within the herd over a 90-day period, based on four consecutive negative polymerase chain reaction (PCR) herd testings every 30 days, or more frequently, of at least 30 suckling pigs at weaning age from 30 different litters. Moreover, there shall be no clinical signs consistent with PRRS observed in the particular breeding herd. Virus detection classifies a herd as unstable.

The aim of this study was to investigate the frequency of PRRSV instability among professional pig breeding farms in northern Germany and the associations between different farm and management characteristics, respectively, and PRRS PCR-status in these herds.

## Methods

### Study herds and data collection

A cross-sectional study was conducted in north-western Germany, the most pig-dense region in Germany. A total of 120 sow herds was included in the study. Inclusion and exclusion criteria were a size of the smallest farrowing group of minimum 12 sows, and the use of an electronic recording system for reproductive performance indicators. Overall, 201 herds were pre-selected. They were member of one out of five different advisory units and they were all contacted first by their (veterinary) advisor. Only herds, where the farmer agreed to participate in the study voluntarily, were included in the final sample. Between July 2011 and November 2012, the herds were examined once, evenly distributed over the whole study period. Herd examination consisted of (1) filling in a paper-based questionnaire on production and management parameters in personal interviews with the farmers (all parameters are listed in an additional file, separately for numerical variables (Additional file 1) and in categorical variables (Additional file 2); (2) taking blood samples from 30 suckling pigs from 10 different litters (3 piglets per litter/17–21 days of age/all randomly chosen) by puncture of the *vena jugularis externa*. Blood samples were taken to the laboratory (Field Station for Epidemiology) and examined for PRRSV with a commercially available real-time PCR kit (Tetracore Inc., Rockville, USA) in pools, each containing the 3 samples from the suckling pigs of the same litter. Due to limited time and financial resources, the sampling protocol of sampling all herds once was not strictly in line with Holtkamp’s definition of PRRSV stability [10], requiring various consecutive herd testings. Thus, whereas a positive PCR result clearly classifies a herd as unstable, a lack of PRRSV detection at sampling does not proof that a herd is stable. For this reason, in the course of this manuscript we would like to use the term “PCR negative” to describe the absence of virus circulation in the herd at the time of sampling.

### Data analysis

Data from questionnaires and laboratory results were stored in a spreadsheet program (Microsoft^®^ Excel^®^ 2016, Microsoft Corporation, Redmond, WA, USA); statistical analyses were conducted in R studio for R version 3.0.1 (R Core Team 2013, Vienna, Austria).

Descriptive statistics were calculated for all variables (indicated in Additional files 1 and 2). The herd status as result of PCR testing was defined as the main outcome variable. A herd was classified as PCR positive if at least one blood sample was tested positive by PCR; otherwise, it was classified as PCR negative. Univariable associations between all variables and PCR status were tested with χ^2^ test or Fisher’s Exact test for categorical variables and Mann/Whitney-U test for numerical variables. Categorical variables exhibiting very low numbers in certain categories were recoded (summing up two categories into one new one). An example is the production rhythm, where only few herds had a 2-weekly or 5-weekly rhythm, so that the different weekly rhythms were summarized in the two categories “1- or 2-weekly” and “3-, 4- or 5-weekly”. Numerical variables were checked for linear relationship with the outcome variable by creating scatterplots and adding smoothed lines (function *lowess*). If this was not fulfilled, variables were categorized following biologically or otherwise plausible boundaries (e.g. suckling period up to vs. more than the legally required 21 days), or according to three equal quantiles (herd size, time interval between purchase of gilts). All variables showing a *p*-value of ≤ 0.1 in the univariable analysis were treated as candidate variables for a multivariable logistic regression model, and tested for associations among them (Spearman correlation coefficient for numerical variables and Cramer’s V/Phi-coefficient (function *assocstats* from the R *vcd* package). In the case of two variables showing a correlation coefficient (R) of 0.5 or higher, the biologically more plausible variable was retained. Additionally, the full model containing the 86 complete observations for all candidate variables was checked for multi-collinearity by calculating the general variance inflation factor (function *vif* of the *car* package). Based on the full model, a step-wise backward selection process was conducted using R function *step (direction* =*“both”)*, based on Akaike’s as well as Bayesian Information Criterion (AIC and BIC). In addition, a manual backward selection based on Likelihood Ratio-Test (LRT, significance level: p ≤ 0.05) was performed on the full model to check if this would result in the same final model. The LRT was used as well to compare the resulting two different final models. Two-way Interactions were tested between all variables of the final models.

## Results

Of the 120 herds in this study, PRRSV was detected in 32 herds, whereas 88 herds were tested PCR negative. Positive results were distributed evenly over the study period; no influence of the season was seen. The median herd size over all herds was 240 sow places or 2091 pigs (excluding suckling pigs) in total. Most of the herds were farrow-to-finish herds (n = 86), the remaining herds were piglet producers selling nursery pigs (n = 29) or suckling pigs (n = 5). Further herd characteristics and univariable comparisons between PCR positive and PCR negative herds are displayed in Additional files 1 and 2.

The results of multivariable logistic regression analyses (full and final models) are presented in Table 1. Due to multi-collinearity with several other variables (e.g. farrowing rhythm or presence of external staff), herd size was not included in the full model, because it was deemed the less directly effect-related variable. Alternative full models including herd size alone and in conjunction with the highly correlated variables both led to the same final model after variable selection.

**Table 1:** Results of full model, final model 1 (automated step-wise backward selection based on akaike information criterion AIC) and 2 (manual backward selection): coefficients (Coeff.), standard error (SE) and p-value of the wald test (Wald) for each separate category of a variable as well as likelihood ratio-test (LRT) indicating the p-value of the overall variable (final models only)

Variable/category	Full model			Final model 1				Final model 2			
	Coeff.	SE	Wald	Coeff.	SE	Wald	LRT	Coeff.	SE	Wald	LRT
(Intercept)	− 1.00	2.59	0.70	− 1.10	1.00	0.27		− 1.75	0.91	0.05	
Suckling period (days)											
≤ 21											
≥ 22	− 1.53	1.02	0.13	− 1.37	0.80	0.09	0.08	− 1.49	0.71	0.04	0.03
Sows—genetic											
Danbred											
BHZP or Topigs	− 1.27	1.02	0.22								
PIC, Hülsenberger, Hermitage, other	− 1.97	1.08	0.07								
Time interval between purchase of gilts (weeks)											
< 9											
9	− 1.63	1.38	0.24	− 0.72	0.95	0.45	0.06				
10–52	− 2.55	1.10	0.02	− 1.82	0.84	0.03					
Time interval in which sows are sent to slaughter (weeks)	0.08	0.29	0.79								
Time interval between pick-up of carcasses (days)	0.02	0.10	0.83								
Distance between sow barn and pick-up location for carcasses (m)	− 0.02	0.01	0.09	− 0.02	0.01	0.03	0.01	− 0.01	0.01	0.06	0.03
Quarantine/acclimatization unit—number of gilts per compartment (n)	0.01	0.03	0.71								
Pig density in county											
Low											
High	1.48	0.89	0.10								
Number of other pig farms within 1000 m distance											
≤ 1											
≥ 2	3.94	1.33	< 0.01	2.88	0.92	0.00	< 0.001	2.29	0.81	0.01	< 0.001
Boar semen—purchase from PRRSV unsuspicious boar stud											
No											
Yes	− 0.83	0.81	0.31								
Hygiene lock											
No clear separation											
Clear separation	0.88	1.21	0.47								
Employment of external staff											
No											
Yes	1.29	0.86	0.13	1.70	0.74	0.02	0.01	1.51	0.65	0.02	0.01
Farrowing rhythm											
1- or 2-weekly											
3-, 4- or 5-weekly	− 0.90	0.85	0.29	− 1.06	0.66	0.11	0.10				
Suckling pigs—age at ear tagging (day)											
1st											
≥ 2nd	− 0.96	0.86	0.26								
Suckling pigs—2nd standard antibiotic treatment											
No											
Yes	− 0.42	0.82	0.61								
Sows—vaccination against PRRSV											
No											
Yes	− 0.09	1.32	0.94								
AIC	94.9			81.8				84.0			

*BHZP* “Bundes Hybrid Zucht Programm”, German commercial provider of hybrid sows, *PIC* “Pig Improvement Company”, North American commercial provider of hybrid sows

The final model after manual backward selection (final model 2; automated stepwise selection based on BIC resulted in the same final model) contained four variables significantly associated with herd status (based on LRT).

Herds working with a suckling period > 21 days had an odds ratio (OR) of 0.23 for being PCR positive thus unstable, compared to herds with a suckling period of ≤ 21 days [95%-confidence interval (CI) 0.05–0.88]. Every additional meter distance between the cadaver collection site and the actual sow barn decreased the chance of being unstable (OR 0.99, CI 0.98–0.99). Herds with ≥ 2 pig herds in their vicinity (1000 m radius) had a 9.91-fold higher chance of being unstable than herds with maximum one other herd in their vicinity (CI 2.35–60.8). The OR of being unstable for herds with external employees (people employed for taking care of the pigs, i.e. not belonging to the herd owning family or household) compared to herds without external employees was 4.52 (CI 1.34–17.43).

The final model after automated step-wise backward selection based on AIC (final model 1) included the same variables as final model 2: the distance between sow barn and pick-up location for carcasses (OR 0.98, CI 0.97–0.99), the number of farms in their vicinity (OR 17.78, CI 3.52–138.06), presence of external employees (OR 5.46, CI 1.39–26.24) and the suckling period (OR 0.25, CI 0.05–1.16) remained significant. Two additional variables were the time interval between purchase of gilts—an interval of 9 weeks had an OR of 0.49 (CI 0.059–2.79), an interval of 10 or more weeks an OR of 0.16 (CI 0.03–0.75) compared with smaller intervals—and a 3- or more-weekly farrowing rhythm had an OR of 0.35 (CI 0.09–1.23). Although the latter three variables were not significant according to the set α-level, the LRT for comparison of the two final models gave a p-value of 0.04. This, together with the lower AIC, indicated a superiority of the larger final model 1 (automated step-wise backward selection) to explain the observed data. No significant interactions were found between any two variables of the final models.

## Discussion

This study aimed to investigate the frequency of PRRSV instability among professional pig breeding farms and to assess risk factors for PRRSV instability in German sow herds. Knowledge about PRRSV status in terms of virus circulation is crucial for both, farmers and veterinary practitioners, when planning actions for controlling PRRSV infections in pig herds by different prevention measures [10]. Since the presence of risk factors can influence this status, and also the efficiency of various control measures, the assessment of these risk factors in individual herds needs appropriate attention. The presented investigation is the first cross-sectional study describing the prevalence of ‘PRRSV unstable herds’ and risk factors in professional pig farms in Germany, which are also typical for other North European countries with intensive production systems, e.g. Denmark and The Netherlands. Due to time and financial constraints, the applied sampling protocol to assess the herd status by testing it at one point in time differed from Holtkamp’s proposed methodology of four repeated testings over 90 days [10]. Therefore, it cannot be stated for all herds tested negative in this study that they were actually PRRSV stable according to Holtkamp. However, with the high number of animals sampled per herd, we assume that an overall negative PCR result gives sufficient evidence of lack of virus circulation in weaning-age pigs in this herd, at least at the time of sampling. On the other hand, from this result, it cannot be concluded that a herd is truly PRRSV negative or provisionally negative as defined by Holtkamp categories IV and III, nor is a distinction between stable (category II) and (provisionally) negative possible. This would require a more intense sampling protocol and an evidenced outbreak history, which was not available for study herds, since PRRSV is endemic in the region for years and vaccine coverage against PRRSV is high. However, for our main purpose of the study, which was to assess virus circulation in the breeding herd, i.e. suckling pigs, this distinction is deemed to have limited relevance.

A sample of herds that have been selected from an institutional database was examined in this study. The total herd size was neither a primary selection nor inclusion criterion, but due to the minimum number of 12 sows per farrowing group that was requested for the purpose of obtaining all samples in 1 day (i.e. at least 10 l in the farrowing area expected), the average herd size of farms herein was considerably high (> 300 sows/herd). At the same time the average number of sows in German breeding farms was 211 (Source: http://www.destatis.de), in Dutch breeding farms 240 (Source: http://www.thepigsite.net) and in Danish breeding farms 422 (Source: http://www.lf.dk). Therefore, the study population examined here is size-wise considered as being representative for the target population, i.e. the intensive pig farming systems in northern Europe.

The sample size used in this study was derived from the ‘within-herd’ sampling protocol in order to assess the PRRSV status’ according to Holtkamp et al. [10]. The protocol per sampling was slightly modified as well, taking samples from three piglets per litter instead of only one, and, thus, not perfectly matching the recommendation, but close to. However, this compromise was necessary considering the smaller herd size of European sow farms compared to those in the USA, where the sampling protocol according to Holtkamp et al. [10] had been developed. Farms accommodating approximately 300 sows (average in this study) usually maintain farrowing groups of 15 (1 week farrowing rhythm) to a maximum of 45 sows (3 week farrowing rhythm), which precludes the accessibility of 30 different litters every time in every farm.

Statistical analyses and the modelling approach used herein were conducted following a standard, i.e. well-established, protocol (variable selection, selection of final model, etc.). Two different approaches were used to obtain the final model after multivariable logistic regression, in order to check the robustness of the results. The fact that both final models contained the same set of statistically significant variables confirmed this. Nevertheless, the final model after automated step-wise backward selection based on AIC (final model 1) contained two additional variables. Although these did not exhibit p-values below the significance level of 0.05, they apparently help to explain parts of the overall variation in the data, as can be deducted from the better AIC value of this final model compared to the other one.

The variable ‘number of sows’ was not considered in the final multivariable models in spite of being known as a common confounder in herd level risk factor studies [11] due to the very strong association with other variables (particularly total herd size and staff). Alternative full models built in the modelling process that included this variable, yielded exactly the same final model as described above; herd size had always dropped out as non-significant. Concerning the possible causal pathway, we acknowledge that herd size itself can have a direct effect on virus circulation, given that in larger herds the possibility of having naïve subpopulations of, e.g. gilts is higher. Nevertheless, we think that the biggest proportion of its influence is rather indirect: in the study region, bigger herds tend to be run more professionally, with a higher chance for external employees, they have a different farrowing rhythm, often wean piglets earlier or have shorter intervals for the purchase of gilts; factors that are assumed to have a more direct influence on the herd status. Given the fact that the influence of herd size in risk factor studies has been described elsewhere, it was decided to focus on the other variables.

The significant protective effect of a longer suckling period is in contrast to the idea of reducing the risk of vertical pathogen transmission by keeping this period, i.e. time under risk, as short as possible. This principle of protecting piglets from sow’s infection by early separation and thereby reducing the time under exposure has been successfully implemented with the ‘segregated early weaning’ to combat *Mycoplasma hyopneumoniae* transmission from sows to their offspring [2]. However, in the present study, results indicate that any risk of PRRSV transmission from the dam to their offspring is not increasing over time, but decreasing. Similar observations of a reduced risk of PRRSV infection with longer suckling periods have been made in another study [12], whereas elsewhere the length of this period was not linked in any way to PRRSV infection in pigs from these litters [13]. It can be hypothesized that no increased risk of infection is observed here, because sows (in stable herds) transfer neutralizing and non-neutralizing PRRSV specific antibodies to suckling pigs. The neutralizing PRRSV specific antibodies are protecting them from corresponding infection up to 5 weeks of life [14]. The non-neutralizing antibodies are suspected in potentially assisting viral clearance in already infected animals by Antibody-dependent cellular phagocytosis (ADCP), Antibody-dependent complement-mediated cytotoxicity (CDC) and other mechanisms, which have been described for immune reactions directed to human immunodeficiency virus [15]. Such a phenomenon would explain a decreased PRRSV positivity in piglets from herds with a longer suckling period. Piglets in the present study were aged 17–21 days at sampling, whereby it can be anticipated that piglets in herds with a longer suckling period tended to be a few days older than piglets in herds with only 3 weeks of suckling period, where the actual weaning age is often 20 days of age or even less. The transfer of neutralizing maternally derived antibodies and their protective effect in receiving pigs as assumed in this study is in contrast to the epidemiology of *M. hyopneumoniae* infection, where antibodies do not prevent from infection at all. Another hypothesis could be that piglets weaned after a shorter suckling period are less resistant, contract PRRSV infection more easily after weaning and shed higher amounts of virus. Since in the most predominant farming systems in the investigated region sows and weaned pigs are kept at the same location, these weaned pigs might destabilize the whole sow herd. Finally, the possibility that farms could have reduced the suckling period in an attempt to control PRRSV infection does not seem likely because herds mostly were not aware of any clinical impact of PRRSV.

The influence of the proximity of neighbouring herds, i.e. number of other pig herds within a 1000 m radius, and of the pig density on the likelihood of the sow herd PRRSV PCR status are indicating an airborne transmission of PRRSV, which has been suggested and described elsewhere [2, 12, 16]. The frequency of such an event is widely unknown and is obviously depending on environmental factors determining the stability of PRRSV outside the host [17]. All study herds were located in an area of Germany, where endemic PRRSV infection of nearly all pig herds has been observed earlier [18] and sampling was conducted during a period of 1 year, thus covering all seasons and various environmental conditions. Therefore, airborne transmissions of PRRSV isolates of different virulence, etc. between herds in this region must be assumed and their occurrence is high likely associated to the distance between the farms, i.e. pig density. Once a new (more pathogenic) PRRSV isolate is successfully transmitted into a pig herd, destabilization is depending on cross-protection induced by the previous PRRSV infection in the pigs [18].

The increasing distance between the barn and the storage bin for dead pigs was observed as being protective in terms of PRRSV instability. This might be related to the content of the bin itself, i.e. pigs that potentially died from PRRS and still harbor PRRSV [18], or the fact that emptying the bin requires a rendering truck coming close to the barn. This rendering truck is usually not clean, because it is used for collection of cadaver from several farms per day. It has been shown that both, the tires of a truck and the boots of the driver, can transmit PRRSV, when no sufficient hygienic precautions are in place [18].

A similar impact on the PRRSV status by a lack in the external biosecurity could be speculated with regards to external employees. There are numerous reports pointing out the importance of an adequate hygiene lock and further requirements for visitors and personnel before entering a pig farm [18, 19]. Considering a higher number of people having regular access to the barns, implies an increased risk of someone not totally fulfilling or even omitting hygiene procedures, thereby lowering both, internal and external biosecurity. Apart from the numerical increase of people entering the farm, it is well known that farm employees are less aware of the necessity and the correctness of best hygiene practice as compared to farm owners [20]. Taking these potential risks together it is conclusive that pig farms with external employees are more prone to the entry of new isolates of PRRSV and within-herd circulation of PRRSV, both leading to PRRSV instability.

The factor ‘Time interval between purchase of gilts: 9 weeks’ indicated a trend in the Final Model 1, but was not significantly associated with the PRRSV status. The same applies to the factor ‘Farrowing rhythm: 3-, 4- or 5-weekly’. Regarding the latter one it can be hypothesized that farms operating with a 1- or 2-week farrowing rhythm have a less strict separation of piglets of different age and, therefore, a higher chance of within herd transmission of pathogens. In farms with a 3-, 4- or 5-week rhythm, the older group is already or nearly weaned, when new piglets are born, thereby preventing a transfer of pathogens by staff or other vectors between the different farrowing compartments for the corresponding farrowing group.

## Conclusions

The PRRSV status could be determined with an adopted sampling and testing protocol that suits smaller pig herds as compared to the USA. In 27% of the study herds PRRSV was detected in suckling pigs indicating PRRSV instability. The PRRSV status in German sow herds of average size relies on external and internal biosecurity (pig density in the area, rendering management, external employees), as well as on farm specific management factors (suckling period). Before or while taking actions to control PRRS by known measures, farmers and veterinarians should also eliminate risk factors in the herd and select appropriate measures accordingly.

## Additional files

**Additional file 1.** Descriptive statistics for numerical variables: mean, standard deviation (SD), median (Med), minimum (Min), Maximum (Max) and test result of the Mann/Whitney-U Test (p-value) between PRRSV-positive (pos), i.e. unstable, and PRRSV-negative (neg) herds.

**Additional file 2.** Categorical variables: number of herds in PRRSV positive (pos) and PRRSV negative (neg) herds per category and result of Chi square/Fisher’s Exakt test (p-value).
