# Supplementary material for: Cross-sectional study on risk factors for Porcine Reproductive and Respiratory Syndrome virus sow herd instability in German breeding herds
Source: Acta Vet Scand. 2018 Sep 19;60:57. doi: 10.1186/s13028-018-0411-7 (PMC6146660; doi:10.1186/s13028-018-0411-7)
Supplement: Supplementary file 1 — Additional file 1. Descriptive statistics for numerical variables: mean, standard deviation (SD), median (Med), minimum (Min), Maximum (Max) and test result of the Mann/Whitney-U Test (p-value) between PRRSV-positive (pos), i.e. unstable, and PRRSV-negative (neg) herds. [file 13028_2018_411_MOESM1_ESM.docx]

Additional file 1: Descriptive statistics for numerical variables: mean, standard deviation (SD), median (Med), minimum (Min), Maximum (Max) and test result of the Mann/Whitney-U Test (p-value) between PRRSV-positive (pos) i.e. unstable, and PRRSV-negative (neg) herds

| **Variables** | **PRRSV** | **Herds (n)** | **Mean** | **SD** | **Med** | **Min** | **Max** | **p-value** |
| --- | --- | --- | --- | --- | --- | --- | --- | --- |
| ***Herd size*** |  |  |  |  |  |  |  |  |
| Number of pigs in total (n) | pos | 32 | 2988.3 | 2070.79 | 2351 | 684 | 11654 | 0.05 |
|  | neg | 88 | 2297.7 | 1565.59 | 1989.5 | 154 | 10190 |  |
| Number of sows (n) | pos | 32 | 401.3 | 235.16 | 320 | 100 | 900 | 0.004 |
|  | neg | 88 | 309.9 | 315.85 | 210 | 110 | 2000 |  |
| Number of weaning pigs (n) | pos | 31 | 1589 | 1086.33 | 1300 | 300 | 4500 | 0.01 |
|  | neg | 84 | 1168 | 1097.97 | 900 | 300 | 8000 |  |
| ***External biosecurity*** |  |  |  |  |  |  |  |  |
| Distance to the closest pig herd within 1000m radius (m) | pos | 31 | 437.1 | 241.86 | 500 | 100 | 900 | 0.27 |
|  | neg | 76 | 380.1 | 247.6 | 300 | 20 | 1000 |  |
| Distance to the closest public road (m) | pos | 32 | 530 | 494 | 335 | 20 | 2000 | 0.43 |
|  | neg | 88 | 481.7 | 584.5 | 300 | 10 | 3000 |  |
| Annual replacement rate (%) | pos | 30 | 40.1 | 8.25 | 39.4 | 28.1 | 61.2 | 0.28 |
|  | neg | 77 | 42 | 9.54 | 42.3 | 21.6 | 87.2 |  |
| Sows with artificial insemination (%) | pos | 32 | 98.6 | 3.77 | 100 | 80 | 100 | 0.42 |
|  | neg | 88 | 98.9 | 2.05 | 100 | 90 | 100 |  |
| Regular time interval between purchase of gilts (weeks) | pos | 27 | 7.6 | 2.08 | 8 | 4 | 12 | 0.05 |
|  | neg | 79 | 9.5 | 6.32 | 9 | 2 | 52 |  |
| Regular time interval in which sows are sent to slaughter (weeks) | pos | 32 | 2.7 | 1.42 | 2 | 1 | 8 | 0.04 |
|  | neg | 88 | 3.4 | 2.17 | 3 | 1 | 16 |  |
| Regular time interval between sales of weaning pig (weeks) | pos | 19 | 3.1 | 2.81 | 2 | 1 | 12 | 0.18 |
|  | neg | 61 | 3.4 | 2.97 | 3 | 1 | 16 |  |
| Regular time interval between pick-up of carcasses (days) | pos | 32 | 7.1 | 3.25 | 7 | 4 | 21 | 0.06 |
|  | neg | 87 | 9.4 | 5.68 | 7 | 4 | 28 |  |
| Distance between sow barn and pick-up location for carcasses (m) | pos | 32 | 61.6 | 43.93 | 50 | 10 | 200 | 0.09 |
|  | neg | 88 | 110.4 | 216 | 50 | 8 | 2000 |  |
| Regular time interval between farm visits by veterinarian (weeks) | pos | 32 | 3.8 | 3.43 | 3 | 1 | 12 | 0.34 |
|  | neg | 88 | 4.4 | 3.56 | 3 | 1 | 12 |  |
| Regular time interval between farm visits for gestation checks (weeks) | pos | 18 | 2.4 | 0.86 | 2 | 1 | 4 | 0.12 |
|  | neg | 51 | 2.8 | 0.78 | 3 | 1 | 5 |  |
| Quarantine /acclimatization unit - number of gilts per compartment (n) | pos | 28 | 34 | 24.41 | 27.5 | 6 | 100 | 0.02 |
|  | neg | 77 | 22.3 | 15.1 | 16 | 6 | 94 |  |
| Quarantine /acclimatization unit - number of gilts per pen (n) | pos | 28 | 10 | 6.49 | 8 | 1 | 34 | 0.91 |
|  | neg | 78 | 9.8 | 6.2 | 9 | 1 | 47 |  |
| Duration of quarantine /acclimatization (weeks) | pos | 28 | 6.3 | 1.59 | 6 | 4 | 10 | 0.21 |
|  | neg | 79 | 5.9 | 1.46 | 6 | 3 | 10 |  |
| ***Internal biosecurity*** |  |  |  |  |  |  |  |  |
| Insemination centre - number of compartments (n) | pos | 28 | 1.5 | 0.84 | 1 | 1 | 4 | 0.54 |
|  | neg | 69 | 1.3 | 0.53 | 1 | 1 | 3 |  |
| Insemination centre - Number of sow places (all or biggest compartment) (n) | pos | 28 | 72.8 | 49.72 | 58 | 14 | 192 | 0.24 |
|  | neg | 69 | 58.4 | 36.72 | 50 | 18 | 224 |  |
| Gestation barn - number of compartments (n) | pos | 28 | 1.68 | 1.19 | 1 | 1 | 5 | 0.39 |
|  | neg | 69 | 1.52 | 1.12 | 1 | 1 | 6 |  |
| Farrowing unit - number of empty days before restocking | pos | 32 | 2.8 | 2.97 | 1 | 0 | 10 | 0.15 |
|  | neg | 88 | 3.6 | 3.32 | 2 | 0 | 14 |  |
| Farrowing unit – number of compartments (n) | pos | 32 | 7.9 | 4.42 | 7 | 3 | 24 | 0.51 |
|  | neg | 88 | 6.7 | 2.22 | 7 | 1 | 12 |  |
| Farrowing unit – number of sow places per compartment (all or biggest compartment) (n) | pos | 30 | 16.4 | 9.59 | 12 | 6 | 38 | 0.78 |
|  | neg | 77 | 17.3 | 18.5 | 12 | 6 | 144 |  |
| Farrowing unit – arrival of sows before farrowing (days) | pos | 32 | 2 | 0.47 | 2 | 1 | 3 | 0.51 |
|  | neg | 88 | 2 | 0.49 | 2 | 1 | 3 |  |
| Suckling pigs - age at castration (days) | pos | 32 | 4.2 | 1.82 | 4 | 0 | 10 | 0.61 |
|  | neg | 88 | 4.1 | 2.01 | 4 | 0 | 10 |  |
| Nursery unit - number of pigs per compartment (all or biggest compartment) (n) | pos | 31 | 204.5 | 120.11 | 180 | 70 | 700 | 0.74 |
|  | neg | 77 | 211.7 | 138 | 160 | 45 | 660 |  |
| Nursery unit - number of pigs per pen (all or biggest pen) (n) | pos | 30 | 28 | 8.39 | 25 | 15 | 50 | 0.33 |
|  | neg | 71 | 31.5 | 16 | 30 | 8 | 130 |  |
| Nursery unit - number of empty days before restocking | pos | 31 | 1.8 | 0.43 | 2 | 1 | 2 | 0.19 |
|  | neg | 73 | 1.9 | 0.33 | 2 | 1 | 2 |  |
| ***Health and treatments*** |  |  |  |  |  |  |  |  |
| Suckling pigs - age at 1. administration of iron (days) | pos | 32 | 2.4 | 1.78 | 2.5 | 1 | 10 | 0.7 |
|  | neg | 88 | 2.3 | 1.34 | 2 | 1 | 7 |  |
| Suckling pigs - age at 1. standard antibiotic treatment (days) | pos | 31 | 2.8 | 2.07 | 3 | 1 | 10 | 0.48 |
|  | neg | 77 | 3.1 | 2.11 | 3 | 1 | 10 |  |
| ***Reproductive performance (according to sow planner)*** |  |  |  |  |  |  |  |  |
| Return-to-oestrus rate, total (%) | pos | 30 | 9.5 | 4.19 | 8.5 | 3.7 | 23.7 | 0.71 |
|  | neg | 78 | 9.7 | 4.15 | 9.2 | 1.7 | 22.4 |  |
| Return -to-oestrus rate, gilts (%) | pos | 30 | 13.3 | 6.94 | 12.5 | 2.4 | 36 | 0.95 |
|  | neg | 73 | 13.4 | 7.59 | 12 | 0 | 39.2 |  |
| Return -to-oestrus rate, sows (%) | pos | 30 | 8.6 | 4.1 | 7.9 | 2 | 19.9 | 0.71 |
|  | neg | 73 | 8.9 | 4.05 | 7.9 | 1.9 | 20.9 |  |
| Proportion of litters - gilts (%) | pos | 30 | 16.6 | 3.36 | 16.9 | 10.4 | 22 | 0.22 |
|  | neg | 77 | 18.1 | 4.5 | 17.3 | 8.3 | 34.8 |  |
| Proportion of litters - sows (%) | pos | 30 | 83.4 | 3.36 | 83.1 | 78 | 89.6 | 0.21 |
|  | neg | 77 | 81.9 | 4.49 | 82.7 | 65.2 | 91.7 |  |
| Days in gestation (n) | pos | 30 | 115.4 | 0.58 | 115.3 | 114.1 | 116.4 | 0.63 |
|  | neg | 73 | 115.3 | 0.89 | 115.3 | 111.5 | 117.3 |  |
| Age at first farrowing (days) | pos | 29 | 371.2 | 14.65 | 371 | 341 | 402 | 0.97 |
|  | neg | 73 | 372.4 | 17.21 | 370 | 335 | 449 |  |
| Farrowing rate (%) | pos | 30 | 83.2 | 5.01 | 83.6 | 65.6 | 90.4 | 0.77 |
|  | neg | 78 | 83.7 | 5.4 | 83.5 | 68.9 | 95.6 |  |
| Live-born piglets per litter, total (n) | pos | 30 | 13.4 | 1.05 | 13.2 | 12.1 | 16.1 | 0.47 |
|  | neg | 78 | 13.5 | 0.96 | 13.5 | 11.2 | 15.7 |  |
| Live -born piglets per litter, gilts (n) | pos | 30 | 12.9 | 1.15 | 12.8 | 11.2 | 15.3 | 0.53 |
|  | neg | 77 | 13.1 | 1.06 | 12.9 | 10.9 | 15.5 |  |
| Live -born piglets per litter, sows (n) | pos | 30 | 13.5 | 1.05 | 13.4 | 12.1 | 16.6 | 0.61 |
|  | neg | 77 | 13.5 | 0.98 | 13.3 | 11.1 | 15.9 |  |
| Still-born piglets per litter, total (n) | pos | 30 | 9.2 | 2.74 | 8.9 | 3.1 | 14.2 | 0.88 |
|  | neg | 77 | 9.1 | 2.54 | 8.9 | 1.6 | 15.7 |  |
| Still -born piglets per litter, gilts (n) | pos | 30 | 6.6 | 2.47 | 6.2 | 0.9 | 12 | 0.3 |
|  | neg | 73 | 7 | 2.44 | 6.9 | 0 | 13.9 |  |
| Still -born piglets per litter, sows (n) | pos | 30 | 9.6 | 2.82 | 9.4 | 3.6 | 14.9 | 0.99 |
|  | neg | 73 | 9.6 | 2.78 | 9.5 | 1.6 | 16.3 |  |
| Piglets per sow per year, total (n) | pos | 30 | 34.9 | 3.91 | 34.2 | 28.2 | 43.6 | 0.95 |
|  | neg | 78 | 34.6 | 3 | 34.5 | 28.6 | 41.7 |  |
| Live -born piglets per sow per year (n) | pos | 30 | 31.6 | 2.94 | 31.4 | 26.5 | 37.3 | 0.92 |
|  | neg | 78 | 31.5 | 2.43 | 31.5 | 25.5 | 37.6 |  |
| Foster litters (%) | pos | 27 | 2 | 2.76 | 1.1 | 0 | 12.6 | 0.18 |
|  | neg | 69 | 3 | 3.47 | 1.7 | 0 | 13 |  |
| Suckling days (n) | pos | 30 | 22.8 | 3.42 | 21.4 | 19 | 30.1 | 0.01 |
|  | neg | 78 | 24.6 | 2.95 | 25.9 | 19.2 | 29.4 |  |
| Weaned piglets per litter, total (n) | pos | 30 | 11.4 | 0.88 | 11.4 | 9.7 | 13.2 | 0.94 |
|  | neg | 78 | 11.3 | 1.07 | 11.3 | 5.1 | 13.3 |  |
| Weaned piglets per litter, gilts (n) | pos | 30 | 11.7 | 0.8 | 11.7 | 10.1 | 13.3 | 0.43 |
|  | neg | 73 | 12 | 1.29 | 11.8 | 10.2 | 17.9 |  |
| Weaned piglets per litter, sows (n) | pos | 30 | 11.3 | 0.88 | 11.3 | 9.6 | 13.2 | 0.93 |
|  | neg | 73 | 11.3 | 0.81 | 11.2 | 9.6 | 13.5 |  |
| Pre-weaning mortality, total (%) | pos | 30 | 15.6 | 3 | 14.9 | 7.9 | 20.2 | 0.9 |
|  | neg | 78 | 15.8 | 3.63 | 15.9 | 6.6 | 23.8 |  |
| Pre-weaning mortality, gilts (%) | pos | 30 | 14.5 | 3.98 | 14.6 | 8.7 | 23.9 | 0.54 |
|  | neg | 72 | 13.6 | 4.03 | 14.4 | 0.4 | 21.9 |  |
| Pre-weaning mortality, sows (%) | pos | 30 | 15.8 | 3.17 | 15.9 | 7.7 | 21.7 | 0.63 |
|  | neg | 72 | 16.1 | 3.77 | 15.6 | 7.2 | 24.7 |  |
| Weaned piglets per sow per year (n) | pos | 30 | 26.9 | 2.49 | 26.7 | 21.7 | 31.5 | 0.7 |
|  | neg | 78 | 26.7 | 2.15 | 26.7 | 21.9 | 31.1 |  |
| Litters per sow per year (n) | pos | 30 | 2.37 | 0.08 | 2.39 | 2.19 | 2.53 | 0.22 |
|  | neg | 78 | 2.34 | 0.08 | 2.35 | 2.07 | 2.53 |  |
