# Supplementary material for: Cross-sectional study on risk factors for Porcine Reproductive and Respiratory Syndrome virus sow herd instability in German breeding herds
Source: Acta Vet Scand. 2018 Sep 19;60:57. doi: 10.1186/s13028-018-0411-7 (PMC6146660; doi:10.1186/s13028-018-0411-7)
Supplement: Supplementary file 2 — Additional file 2. Categorical variables: number of herds in PRRSV positive (pos) and PRRSV negative (neg) herds per category and result of Chi square/Fisher’s Exakt test (p-value). [file 13028_2018_411_MOESM2_ESM.docx]

Additional file 2: Categorical variables: number of herds in PRRSV positive (pos) and PRRSV negative (neg) herds per category and result of Chi square / Fisher’s Exakt test (p-value)

| **Variables** | **Categories** | **PRRSV pos** | **PRRSV neg** | **p-value** |
| --- | --- | --- | --- | --- |
| ***Herd characteristics*** |  |  |  |  |
| Production type | piglet producer - sale of weaning pigs | 1 | 4 | 0.96 |
|  | piglet producer - sale of nursery pigs | 8 | 21 |  |
|  | farrow-to-finish (fattening of ≤70% of produced piglets) | 7 | 22 |  |
|  | farrow-to-finish (fattening of >70% of produced piglets) | 16 | 41 |  |
| Farm sites | 1 | 16 | 51 | 0.53 |
|  | 2 | 9 | 25 |  |
|  | ≥3 | 7 | 12 |  |
| Sows - genetic (all or majority) | Danbred | 14 | 28 | 0.07 |
|  | BHZP or Topigs | 5 | 33 |  |
|  | PIC, Hülsenberger, Hermitage, other | 13 | 27 |  |
| Sows - more than one genetic (recent change) | yes | 10 | 22 | 0.49 |
|  | no | 22 | 66 |  |
| ***External Biosecurity*** |  |  |  |  |
| Location of farm in county with | high pig density (Cloppenburg, Vechta) | 23 | 38 | 0.01 |
|  | moderate pig density (Emsland, Osnabrück, Diepholz) | 9 | 50 |  |
| Number of other pig farms within 1000 m distance | 0 or 1 farm | 4 | 31 | 0.01 |
|  | ≥ 2 farms | 28 | 57 |  |
| Production type of the closest pig farm within 1000 m distance | farm with with nursery pigs | 11 | 22 | 0.53 |
|  | farm with fattening pigs/boars only | 20 | 53 |  |
| Boar semen – purchase | from one boar stud | 28 | 78 | 0.54 |
|  | from two or more different boar stud | 4 | 10 |  |
| Boar semen – purchase from PRRSV unsuspicious boar stud | yes | 10 | 43 | 0.09 |
|  | no or unknown | 22 | 45 |  |
| Gilts - replacement | replacement with self-bred animals | 3 | 9 | 0.6 |
|  | purchase or purchase and replacement with self-bred animals | 29 | 79 |  |
| Gilts - purchase, PRRSV status of herd of origin | PRRSV unsuspicious | 18 | 47 | 0.81 |
|  | unknown or positive | 11 | 32 |  |
| Boars - replacement | replacement with self-bred animals | 20 | 52 | 0.74 |
|  | purchase | 12 | 36 |  |
| Gilts - delivery to farm | breeder / raiser | 14 | 34 | 0.46 |
|  | commercial carrier | 13 | 44 |  |
| Nursery pigs - collection (sale, transport to different location) | yes | 19 | 61 | 0.24 |
|  | no | 12 | 23 |  |
| Nursery pigs - collection, transport vehicle | own transport vehicle | 9 | 23 | 0.45 |
|  | external transport vehicle (carrier, buyer) | 10 | 38 |  |
| Slaughter sows - collection | own transport vehicle | 5 | 8 | 0.31 |
|  | commercial carrier | 27 | 80 |  |
| Hygiene lock | no clear separation between dirty and clean area | 18 | 64 | 0.09 |
|  | clear separation between dirty and clean area / shower | 14 | 24 |  |
| Consultants have regular access | yes | 6 | 17 | 0.94 |
|  | no | 26 | 71 |  |
| Gestation check service has regular access | yes | 18 | 51 | 0.87 |
|  | no | 14 | 37 |  |
| Employment of external staff | yes | 24 | 46 | 0.03 |
|  | no | 8 | 42 |  |
| Gilts - purchase, quarantine/acclimatization phase | yes | 28 | 79 | 0.27 |
|  | no | 1 | 0 |  |
| Young boars - purchase, quarantine/acclimatization phase | yes | 5 | 20 | 0.4 |
|  | no | 7 | 16 |  |
| Gilts - purchase, quarantine/acclimatization barn | separate site / barn | 19 | 65 | 0.11 |
|  | separate compartment | 9 | 14 |  |
| Gilts - purchase, housing system quarantine/acclimatization barn | all in - all out | 25 | 74 | 0.35 |
|  | continuously | 3 | 5 |  |
| Gilts - purchase, contacts during quarantine/acclimatization phase | none | 8 | 28 | 0.51 |
|  | faeces, placentae, slaughter sows, nursery pigs | 20 | 51 |  |
| Gilts - integration with sows | separate group until farrowing | 6 | 25 | 0.29 |
|  | together with sows | 26 | 63 |  |
| ***Internal biosecurity*** |  |  |  |  |
| Use of the same equipment in various buildings | yes | 13 | 24 | 0.16 |
|  | no | 19 | 64 |  |
| Production / farrowing rhythm | 1- or 2-weekly | 22 | 37 | 0.01 |
|  | 3-, 4- or 5-weekly | 10 | 51 |  |
| Boars - use for breeding | yes | 17 | 45 | 0.85 |
|  | no | 15 | 43 |  |
| Combined breeding and gestation unit | yes | 4 | 19 | 0.2 |
|  | no | 28 | 69 |  |
| Breeding unit - housing system | all in - all out | 5 | 32 | 0.01 |
|  | continuously | 23 | 37 |  |
| Gestation unit - sow grouping | no groups | 18 | 25 | 0.03 |
|  | dynamic groups | 3 | 20 |  |
|  | fixed groups | 7 | 23 |  |
| Farrowing unit - compartment for intermediate farrowings | yes | 16 | 36 | 0.37 |
|  | no | 16 | 52 |  |
| Farrowing unit - housing system | all in - all out, strictly | 28 | 76 | 0.57 |
|  | all in - all out, not strictly | 4 | 12 |  |
| Farrowing unit - cleaning before restocking | yes, strictly | 31 | 83 | 0.49 |
|  | yes, not strictly | 1 | 5 |  |
| Farrowing unit - disinfection before restocking | yes, strictly | 23 | 66 | 0.73 |
|  | yes, not strictly | 9 | 22 |  |
| Farrowing unit - disinfectant | disinfectant brand 1 | 15 | 41 | 0.13 |
|  | disinfectant brand 2 | 9 | 37 |  |
|  | disinfectant brand 3 | 8 | 10 |  |
| Suckling pigs - age at tail docking | 1st day of life | 15 | 32 | 0.24 |
|  | ≥ 2nd day of life | 16 | 56 |  |
| Suckling pigs - teeth grinding | yes | 23 | 53 | 0.24 |
|  | no | 9 | 35 |  |
| Suckling pigs - age at ear tagging | 1st day of life | 16 | 25 | 0.02 |
|  | ≥ 2nd day of life | 12 | 53 |  |
| Suckling pigs - proportion of fostered pigs per farrowing group | ≤ 10 % | 3 | 12 | 0.53 |
|  | ≥ 11% | 29 | 76 |  |
| Suckling pigs - age at weaning (days) | ≤ 23 | 20 | 38 | 0.06 |
|  | ≥ 24 | 12 | 50 |  |
| Nursery unit - cleaning before restocking | yes | 31 | 73 | 0.71 |
|  | no | 0 | 1 |  |
| Nursery unit - disinfection before restocking | yes | 27 | 60 | 0.33 |
|  | no | 4 | 14 |  |
| ***Health and treatments*** |  |  |  |  |
| Sows - induction of farrowing | only rarely, if prolonged gestation | 18 | 48 | 0.73 |
|  | 115./116. day of gestation, if necessary | 3 | 13 |  |
|  | 113./114 day of gestation, standard | 11 | 27 |  |
| Suckling pigs - 2nd application of iron | yes | 16 | 38 | 0.51 |
|  | no | 16 | 50 |  |
| Suckling pigs - 1st standard antibiotic treatment | yes | 31 | 77 | 0.12 |
|  | no | 1 | 11 |  |
| Suckling pigs - reason for 1st standard antibiotic treatment | infection of castration wound / umbilical infection | 27 | 70 | 0.31 |
|  | respiratory / intestinal infection | 4 | 7 |  |
| Suckling pigs - 2nd standard antibiotic treatment | yes | 22 | 42 | 0.04 |
|  | no | 10 | 46 |  |
| Suckling pigs - reason for 2nd standard antibiotic treatment | infection of castration wound / umbilical infection | 9 | 17 | 0.92 |
|  | respiratory / intestinal infection | 13 | 26 |  |
| Nursery pigs - 1st standard antibiotic treatment | yes | 20 | 52 | 0.56 |
|  | no | 11 | 22 |  |
| Nursery pigs - 2nd standard antibiotic treatment | yes | 10 | 24 | 0.97 |
|  | no | 21 | 50 |  |
| Nursery pigs - health issues | no health issues | 4 | 17 | 0.24 |
|  | health issues | 27 | 57 |  |
| Gilts - 1st standard antibiotic treatment during quarantine / acclimatization | yes | 9 | 21 | 0.57 |
|  | no | 19 | 58 |  |
| Gilts - 2nd standard antibiotic treatment during quarantine / acclimatization | yes | 0 | 8 | 0.08 |
|  | no | 28 | 71 |  |
| Sows – vaccination against PRRSV | yes | 26 | 81 | 0.09 |
|  | no | 6 | 7 |  |
| Sows – PRRSV vaccination scheme | mass vaccination, 3, 4 or 5 months interval | 21 | 57 | 0.34 |
|  | according to the status or reproduction (6/60, during gestation etc.) | 5 | 23 |  |
| Sows – vaccination against porcine circo virus 2 (PCV2) | yes | 6 | 11 | 0.39 |
|  | no | 26 | 77 |  |
| Sows – vaccination against swine influenza virus (SIV) | yes | 20 | 52 | 0.74 |
|  | no | 12 | 36 |  |
| Sows – vaccination against *E. coli* or *E. coli/Cl. perfringens* | yes | 6 | 29 | 0.13 |
|  | no | 26 | 59 |  |
| Sows – simultaneous vaccinations | yes | 14 | 29 | 0.28 |
|  | no | 18 | 59 |  |
| Suckling pigs – vaccination against PRRSV | yes | 10 | 19 | 0.27 |
|  | no | 22 | 69 |  |
| Suckling pigs – age (week of life) at vaccination against PRRSV | 1st or 2nd | 8 | 14 | 0.54 |
|  | 3rd of 3th | 2 | 5 |  |
| Suckling pigs – vaccination against PCV2 | yes | 22 | 57 | 0.69 |
|  | no | 10 | 31 |  |
| Suckling pigs – age (week of life) at vaccination against PCV2 | 1st or 2nd | 1 | 4 | 0.57 |
|  | 3rd of 3th | 21 | 53 |  |
| Suckling pigs – PCV2 vaccine | vaccine A | 6 | 10 | 0.32 |
|  | vaccine B | 15 | 45 |  |
| Suckling pigs – vaccination against *M. hyopneumoniae* | yes | 26 | 63 | 0.29 |
|  | no | 6 | 25 |  |
| Suckling pigs – simultaneous vaccinations | yes | 17 | 47 | 0.99 |
|  | no | 15 | 41 |  |
| Nursery pigs - vaccinations during nursery phase | yes | 8 | 23 | 0.59 |
|  | no | 23 | 51 |  |
| Gilts (in quarantine / acclimatization) – vaccination against PRRSV | yes | 31 | 79 | 0.2 |
|  | no | 1 | 9 |  |
| Gilts – number of PRRSV vaccinations | 1 | 6 | 24 | 0.2 |
|  | 2 | 25 | 52 |  |
| Gilts – vaccination against PCV2 | yes | 22 | 49 | 0.2 |
|  | no | 10 | 39 |  |
| Gilts – number of PCV2 vaccinations | 1 | 18 | 36 | 0.33 |
|  | 2 | 4 | 13 |  |
| Gilts – vaccination against SIV | yes | 23 | 58 | 0.54 |
|  | no | 9 | 30 |  |
| Gilts – vaccination against A. pleuropneumoniae | yes | 10 | 21 | 0.41 |
|  | no | 22 | 67 |  |
| Gilts – vaccination against M. pleuropneumoniae | yes | 14 | 30 | 0.33 |
|  | no | 18 | 58 |  |
| Gilts – vaccination against H. parasuis | yes | 5 | 9 | 0.42 |
|  | no | 27 | 79 |  |
